# Blood donation during COVID-19 lockdown and its association with anger and stress: A cross-sectional study from Syria

**DOI:** 10.3389/fsoc.2022.971804

**Published:** 2023-02-02

**Authors:** Ameer Kakaje, Sabina Mansour, Ayham Ghareeb, Osama Hosam Aldeen

**Affiliations:** ^1^Faculty of Medicine, Damascus University, Damascus, Syria; ^2^University Hospital Geelong, Barwon Health, Geelong, VIC, Australia

**Keywords:** anger, blood donation, developing countries, lockdown, stress, public health, health policy

## Abstract

**Background:**

The demand for blood donations in Syria was high as the country has suffered for 9 years from war, and this demand has substantially increased during and after the lockdown from the COVID-19 pandemic. This study aims to assess blood donations in Syria and their association with multiple factors.

**Methods:**

Online questionnaires were distributed to social media groups, with questions related to stress, anger, and COVID-19 distress.

**Results:**

This study included 1,423 participants, of which 899 (63.2%) were women. Only 48.5% have ever donated blood, of which 33.3% donated only once in their lifetime. Not having a good reason to donate blood was the main reason for not donating. Obtaining documents was the main reason for blood donation among people who donated blood (64.8%). Stress, anger, and fear of infection were not associated with blood donation and its patterns. Losing someone close and being endangered by war were associated with more frequent blood donations in contrast to being distressed by war.

**Conclusion:**

COVID-19 distress, stress, and anger were not associated with the decrease in blood donation. Spreading awareness on volunteer blood donation is crucial to combat blood shortage during stressful times.

## Background

Blood donation is an act of solidarity. To donate blood means to save a life as many patients require blood for survival under severe conditions such as trauma, cancer, and many other medical disorders. In most countries, it is a voluntary act that differs widely according to circumstances and the culture of societies.^1^ While blood donation by most people is voluntary and unpaid, others donate only when a family member or a friend needs a “directed donation.” Some countries offer payments in exchange for blood donation (Tissot et al., 2013).

In general, the amount of blood donated is an indicator of the availability of blood in a country. In 2020, 118.5 million blood donations were collected across the globe and 40% of these donations were from high-income countries (World Health Organisation, 2020). Although blood donation demands during the COVID-19 lockdowns might be lower, the gap between demand and donation widened worldwide (Stanworth et al., 2020). Globally, including in Syria, the number of blood donors substantially decreased which put a strain on local blood supplies (Cai et al., 2020; Kakaje et al., 2020). The World Health Organization (WHO) had declared an increasing gap in access to blood between high- and low-income countries. In many countries, there were concerns related to blood donation during COVID-19. First, there were concerns reported by blood collection staff and donors about COVID-19 exposure. Furthermore, as COVID-19 was still new, transmission through blood transfusion, mainly asymptomatic carriers, was still uncertain (Stanworth et al., 2020), especially owing to the long incubation period and the carriers being asymptomatic for a relatively long time (Cai et al., 2020). Changes in blood inventory, processing, and storage were also required as fewer blood donors were available and preplanning was essential (Stanworth et al., 2020). More careful use of blood and its products was also of concern as around 20–30% of blood components were being wasted (Stanworth et al., 2020). Thus, efficient inventory management with a full evaluation of the demands is necessary (Cai et al., 2020). This should be especially important in Syria where it is very hard to obtain blood products (Kakaje et al., 2020).

Blood donation, in general, can be a stressful event. One large systematic analysis found that donating blood is a stressful event and can increase anxiety and anger (Hoogerwerf et al., 2015). Another study found that people who donate blood exhibit better mental and physical scores and lower anxiety compared to others and this effect increases with the increase in the frequency of blood donation (Luckett et al., 2019).

Although there was a reduction in blood demand, in general, as elective surgeries were postponed, implementing social distancing and lockdowns to prevent the spread resulted in a decrease in blood product availability (Al Mahmasani et al., 2021). Furthermore, worldwide, sickness and restrictions caused a stepwise reduction in blood donations. However, sharing the experiences of countries that were in different stages can help prepare transfusion services and meet the demands during the pandemic times (Stanworth et al., 2020).

The 9-year war in Syria had affected all aspects of life as above 80% were under the poverty line, millions suffered from mental distress, and millions had to flee the country and depend on humanitarian assistance (Kakaje et al., 2021a). The reduced ability to earn and provide food was among the major concerns, despite not having a high number of corona cases (Kakaje et al., 2021c). The 10-week full lockdown that was enforced significantly lowered the blood donation numbers, causing huge demands for blood (Kakaje et al., 2020).

In low-income countries, approximately half of the transfusions were performed in children aged 5 years and younger. In contrast, around 75% of the blood transfusions were performed for patients aged 60 years or older in high-income countries (World Health Organisation, 2020). In Syria, considered one of the low-income countries, most blood transfusions were performed in young children and pregnant women (Kakaje et al., 2020). Although Syria has a relatively young population, meaning there is a larger population able to donate blood, unfortunately, this was not the case.

In Syria, a blood donation certificate (or an exemption from it) is mandatory to obtain certain documents from the government and some public institutions, including public universities. For example, when applying to get a copy of their transcript/graduation certificates from a public university, people need to submit their blood donation certificates along with their IDs and other documents. This is the most common way of making people donate blood and its products as many people would not voluntarily donate blood if they do not have to. These certificates also have an expiry date, meaning people might need to donate more than once over the years. However, this method only aggravated the shortage of blood supply during the COVID-19 lockdowns as people had to stay at home and did not require any documents as most institutions were closed anyway (Kakaje et al., 2020).

Before COVID-19, most blood donations were for obtaining documents (Kakaje et al., 2020) from regular volunteer donations mainly for thalassemia patients, or for giving to relatives. Blood donation campaigns were mostly run in universities or workplaces rather than on social media or television as most of the demands were met. However, there are no studies that report on blood donation in Syria before COVID-19. While blood donation for payment is illegal in Syria, in some countries, some people donate blood for free to get their blood tested for blood-borne diseases, but this is not a common practice in Syria.

Unfortunately, during COVID-19, blood donation campaigns failed to reach most of the population due to the lack of experience and the lockdown. Therefore, the majority of the population was unaware of the drastic need for blood donation across Syria. Some hospitals in Syria could obtain only around 30% of their regular blood product needs (Kakaje et al., 2020).

This study, conducted 2 weeks after the termination of the full 10-week lockdown to assess the blood donation patterns, reports different variables that could have affected the patterns such as anger, stress, and distress from fear of being infected. Furthermore, blood donation in Syria was not previously studied as the demands were usually met, which changed during COVID-19, necessitating this study and addressing this topic.

We speculate that as people did not need to donate blood during the lockdown, there was a decrease in blood donations. We also suspect that distress and anger from COVID-19 and the nine-year war affected the blood donation patterns. This is the first study that assesses blood donation in Syria, particularly during the COVID-19 lockdown, and its association with stress or anger. We aim to assess the blood donation practices in Syria and their association with the previous factors.

## Methods

This study was a nationwide cross-sectional study conducted between 8 June 2020 and 17 August 2020. Online surveys in Arabic were distributed among multiple online social groups several times each day and included participants from all across Syria.

Informed consent was obtained from all participants by explaining in detail about the research, its goals, and the assured anonymity of the participants. The ethical aspects of this research were reviewed and approved by the Damascus University Deanship, which represented the leading Ethical Committee of the Damascus University Faculty of Medicine, and whether the principles were in accordance with those embodied in the Declaration of Helsinki. This research received no funding and data can be made available upon reasonable request.

Blood donation questions were mandatory, while other questions were not. No imputation methods were used for the missing data. The eligibility criteria excluded participants younger than 18 years as the law prevents them from donating blood. Any participant who consented to attend the survey and lived in Syria during the lockdown was included.

We assessed the demographics and indirectly inquired about the socioeconomic status as it is inconvenient to ask about the monthly salaries in Syria and there is no valid questionnaire to use for the Syrian community (Kakaje et al., 2021a). We inquired whether the place of living was rented, whether they were living in an urban or rural area, monthly income adequacy, and educational level. Other questions that were related to demographics, COVID-19, and blood donation are listed in Tables 1, 2.

**Table 1:** Characteristics of responders and their responses to COVID-19 questions.

**Characteristic**	**Frequency**	**Percentage %**	**Characteristic**	**Frequency**	**Percentage %**
**Gender**			Al Hasakah	10	0.7
Male	524	36.8	Homs	241	16.9
Female	899	63.2	Hama	78	5.5
**Change place of living due to war**			Latakia	124	8.7
No	876	62.3	Tartus	101	7.1
Within the same city	226	16.1	**House that you currently live in**		
To another city	267	19.0	Owned	933	65.7
Both	36	2.6	Rented or given by the government	375	26.4
**Distress from war noises**			Living in friends/relatives house	112	7.9
No	216	15.3	**Smoking cigarette or shisha**	
Yes	1,200	84.7	No	596	42.0
**Being a student**			Regular shisha smoker	180	12.7
No	520	36.7	Regular cigarette smoker	248	17.5
In a school	46	3.2	Smoke both regularly	42	3.0
In a university of high institute	851	60.1	Smoker but not regularly	353	24.9
**Living in**			**Monthly income adequacy**		
Countryside	264	18.6	It cannot cover buying the essentials	334	23.6
City	1,154	81.4	It is only enough for essentials from food and drink	855	60.4
**Life Endangered from war**			It is enough for essentials and other things	226	16.0
No	170	12.0	**Education**		
Yes	971	68.3	Elementary	4	0.3
Not sure	280	19.7	Until grade 9	11	0.8
**Losing someone close due to war**			High school	208	14.6
No	649	45.8	University, or high institute	1,016	71.4
Yes	769		Master or higher	184	12.9
**Governorates**			**Characteristic**	**Mean**	**Std. deviation**
Damascus	523	36.8	Age	25.3	6.5
Rif-Dimashq	172	12.1	Stress score	17.8	10.3
Aleppo	96	6.7	DAR-5 score	11.7	4.4
Daraa	16	1.1			
As Suwayda	42	3.0			
Qunaitra	5	0.4			
Idlib	10	0.7			
Deir ez-Zur	2	0.1			
Ar Raqqah	3	0.2			

**Table 2:** Characteristics of COVID-19 worries and blood donation responses.

**Characteristic**	**Frequency**	**Percent %**	**Characteristic**	**Frequency**	**Percent %**
**How worried have you been from COVID-19 in the last 2 weeks from:**			**What is your main reason for donating blood lately?**		
1. Being infected^*^			For a medical reason	35	5.1
Not at all	355	25.8	To help others	163	23.9
Slightly	586	42.6	For a document	442	64.8
Moderately	304	22.1	For thalassemia patients	36	5.3
Very	130	9.5	As I believe it is good for my health	6	0.9
2. Friends or family being infected^*^			**The usual number of times for blood donating a year?**		
Not at all	107	7.7	Less than one time	295	43.8
Slightly	340	24.6	One time	276	40.9
Moderately	355	25.7	Two time	69	10.2
Very	580	42.0	Three and more	34	5.0
3. That you get infected from commuting or the crowds^*^			**Can you estimate the total number of time you donated blood?**		
Not at all	112	8.2	One	226	33.3
Slightly	228	16.6	Two	145	21.4
Moderately	401	29.2	Three	107	15.8
Very	630	46.0	Four	66	9.7
**Do you recall seeing blood donation campaigns?**			Five and more	134	19.8
No	604	42.5	**Was your blood donation affected by?**		
Yes, on social media for blood donation	575	40.5	Not having a reason or a motive	112	17.2
Yes, on social media for thalassemia patients	66	4.6	Blood donation campaigns	52	8.0
Yes, I recall seeing both	175	12.3	Quarantine and COVID-19-related distress	160	24.5
**Have you ever donated blood?**			The blood-donation center was not close to where I live	109	16.7
No, I have never donated blood	478	33.6	Other	219	33.6
No as I have used medical certificate	254	17.8			
Yes, I have donated	665	46.7			
Yes, I am a frequent blood donator for thalassemia patients	26	1.8			
**What is your reason for not donating before COVID-19?**					
I have no reason	511	45.4			
I feel light-headed from seeing blood/needle	208	18.5			
I have a medical reason	283	25.2			
Afraid from acquiring COVID-19 from the hospital	14	1.2			
I do not like to	55	4.9			
It is of no benefit for me	38	3.4			
It was difficult for me to leave the house or it is far	16	1.4			

* Cronbach's Alpha for these three questions is 0.73.

To check if stress or anger lately affected blood donation patterns, we used a validated and reliable Arabic questionnaire that was extracted from the Depression Anxiety Stress Scale-21 (DASS-21) (Ali et al., 2017). DASS-21 has seven questions about stress with four answers rated from 0 to 4, with 0 for answering “Did not apply to me at all—Never” and 4 for answering “Applied to me very much, or most of the time—Almost Always.” We also used an Arabic version of the Dimensions of Anger Reactions 5 (DAR-5) scale to assess problematic anger and its effects on social functioning which is reliable and was used previously on teenagers and adults online in Arabic (Forbes et al., 2004, 2014; Kakaje et al., 2021b). DAR-5 is a screening method that consists of five items with five answers, scoring from 1 to 5 each with the score ranging from 5 to 25. We used the scores of these scales and not the cutoff points as it would be easier for comparison.

DAR-5 and DASS-21 were originally developed in English and validated in Arabic. However, we used Arabic to ask direct questions about distress from COVID-19 and blood donation without translating from other scales.

We used the IBM SPSS software version 26 for Windows to analyze the data. The chi-square and the one-way analysis of variance (ANOVA) were used and two-tailed *p* < 0.05 were considered statistically significant. We used Cronbach's alpha for reliability and Bonferroni correction to correct the *p*-values with multiple tests.

## Results

### Main findings

Overall, 1,611 participants clicked on the link to participate in the study, but 141 did not consent and 47 did not complete the questionnaire and withdrew. However, the number of people who saw the post but did not click or join is unknown. The study included 1,423 participants, of which 524 (36.8%) were men and 899 (63.2%) were women. The mean age was 25.3 years. The characteristics of the participants are listed in Table 1. Furthermore, only 478 (33.6%) participants did not donate blood without having a medical reason to not donate. Individual responses to each of the COVID-19 and blood donation questions are listed in Table 2.

Men donated blood more frequently than women (*p* < 0.001). The other characteristics of DAR-5 and stress scores are listed in Table 3 using one-way ANOVA. Although not donating blood out of fear of acquiring COVID-19 or because it was hard or the center was far had higher stress scores (*p* = 0.012), the number of participants in these two categories were only 30 (2.6%). Therefore, the majority were not affected. In addition, upon using Bonferroni correction, this result was not significant. Cronbach's alpha for the DAR-5 questions was 0.825 and Cronbach's alpha for stress questions was 0.866.

**Table 3:** Demonstrates anger and stress scores with each blood donation response.

**Characteristic**	**DAR-5**			**Stress**		
	**Mean**	**Std. dev**.	***p***	**Mean**	**Std. dev**.	***p***
**Have you ever donated blood?**						
No, I have never donated blood	11.99	4.42	0.161	18.29	10.43	0.159
No as I have used medical certificate	11.80	4.56		18.48	10.10	
Yes, I have donated	11.44	4.25		17.08	10.06	
Yes, I am a frequent blood donator for thalassemia patients	10.96	4.35		17.17	12.41	
**What is your reason for not donating before COVID-19?**						
I have no reason	11.64	4.52	0.557	17.08	9.99	0.012^#^
I feel light-headed from seeing blood/needle	11.78	4.02		18.52	10.02	
I have a medical reason	11.94	4.50		19.61	10.61	
Afraid from acquiring COVID-19 from the hospital	11.85	5.19		19.85	11.53	
I do not like to	12.93	5.14		17.67	10.86	
It is of no benefit for me	12.37	4.01		18.43	8.45	
It was difficult for me to leave the house or it is far	11.73	4.17		24.00	11.90	
**What is your main reason for donating blood?**						
For a medical reason	10.93	4.13	0.739	18.93	12.62	0.090
To help others	11.09	4.16		15.09	10.27	
For a document	11.56	4.32		17.60	9.77	
For thalassemia patients	11.11	3.89		17.65	10.78	
As I believe it is good for my health	11.50	4.18		17.00	9.01	
**The usual number of times for blood donating a year?**						
Less than one time	11.51	4.41	0.020	17.85	9.85	0.018
One time	11.84	4.22		17.46	10.11	
Two time	10.37	3.46		13.56	10.02	
Three and more	10.00	4.26		15.80	11.51	
**Can you estimate the total number of times you donated blood?**						
One	11.62	4.57	0.756	17.44	10.30	0.025
Two	11.61	4.10		18.65	10.88	
Three	11.32	4.12		16.56	8.77	
Four	10.90	3.65		18.00	9.03	
Five and more	11.26	4.24		14.67	10.21	
**Was your blood donation affected by?**						
Not having a reason or a motive	11.17	4.50	0.216	15.78	8.69	0.063
Blood donation campaigns	10.25	4.09		14.50	9.87	
Quarantine and COVID-19-related distress	11.78	4.33		18.75	10.74	
The blood-donation center was not close to where I live	11.47	4.04		17.24	9.57	
Other	11.60	4.16		17.22	10.50	

One-way ANOVA was used in this table.

# Statistical significant results using Bonferroni correction which was p = 0.008 in this table.

Blood donation and reasons to do it were affected by educational level (*p* < 0.001) and whether being a student or not (*p* < 0.001) but not by monthly income adequacy, living in a rented house, or living in the city or the countryside (*p* > 0.05). All previous factors were not associated with not donating blood because of the lockdown or the center being far or hard to reach (*p* > 0.05). The chi-square test was used in the previous variables. The association of blood donation and the reasons for other variables are listed in Table 4.

**Table 4:** Blood donation questions with different variables.

**Characteristics**	**Have you ever donate blood**			Main reason for donating blood?^*^		
	**No**	**Yes**	***p***	For another reason^*^	**For a document**	***p***
**Age [M (SD)]**	23.8 (5.2)	26.8 (7.3)	<0.001^#^	26.2 (6.1)	27.6 (8.2)	0.016
**Gender**						
Male	106	418	<0.001^#^	122	236	0.009
Female	626	273		83	179	
**How worried have you been from COVID-19 in the last 2 weeks from**						
**1. Being infected**						
Not at all	172	183	0.115	60	115	0.037
Slightly	292	294		81	193	
Moderately	167	137		51	79	
Very	76	54		9	40	
**2. That you get infected from commuting or the crowds**						
Not at all	43	64	0.029	20	41	0.980
Slightly	173	167		49	111	
Moderately	175	180		56	116	
Very	319	261		77	162	
**Change place of living due to war**						
No	444	432	0.674	120	286	0.377
Within the same city	116	110		34	71	
To another city	144	123		43	70	
Both	21	15		5	9	
**Distress from war noises**						
No	80	136	<0.001^#^	45	86	0.443
Yes	650	550		158	354	
**Being a student**						
No	214	306	<0.001^#^	84	204	0.164
In a school	37	9		119	229	
In a university of high institute	478	373		1	8	
**Living in**						
Countryside	141	123	0.503	37	79	0.945
City	590	564		167	362	
**Life endangered from war**						
No	86	84	0.013	24	55	0.859
Yes	479	492		149	312	
Not sure	166	114		32	75	
**Losing someone close due to war**						
No	366	283	0.001^#^	71	197	0.015
Yes	364	405		134	243	
**Education**						
Elementary	2	2	<0.001^#^	2	0	0.006
Until grade 9	4	7		5	2	
High School	128	80		23	50	
University, or high institute	539	477		148	298	
Master or higher	59	125		27	92	
**House that you currently live in**						
Owned	482	451	0.134	125	295	0.171
Rented or given by the government	201	174		54	112	
Living in friends/relatives house	48	64		25	35	
**In regards to personal protection availability from COVID-19**						
I did not use any of them	107	130	0.099	35	87	0.629
I found them easy to obtain	258	230		63	150	
I found it hard to buy hand sensitisers	89	63		21	36	
I found it hard to obtain gloves and face masks	120	124		38	82	
It was hard to buy all	149	136		46	83	
**Smoking cigarette or shisha**						
Not smoked or very rarely smoker	353	243	<0.001^#^	62	168	0.062
Regular shisha smoker	92	88		23	61	
Regular cigarette smoker	79	169		61	95	
Smoke both regularly	16	26		6	20	
Smoker but not regularly	191	162		53	96	
**Monthly income adequacy**						
It cannot cover buying the essentials	177	157	0.799	48	91	0.716
It is only enough for essentials from food and drink	437	418		123	275	
It is enough for essentials and other things	114	112		33	74	

Other reasons included all other reasons in Table 2 except medical reasons which were excluded.

* We have excluded participants who had a medical reason not to donate blood.

# Statistical significant results using Bonferroni correction which was 0.004 in this table.

### Other variables

Most variables that were not mentioned in the tables did not have a significant impact as the number of participants who answered these questions was low even when the *p*-value was significant and are not related to the main aim of the study. Therefore, a few results that might be relevant are mentioned in the following paragraphs.

When using chi-square and applying Bonferroni correction, not donating blood out of fear of COVID-19 was not significantly different between the two genders. However, declaring that the medical center was far or hard to reach was more common among males (*p* = 0.013), which is still not significant upon using the Bonferroni correction. Furthermore, males donated blood more frequently to get documents than females who donated to help people more often (*p* = 0.009) which is not significant. Participants from Damascus, Tartus, and Rif-Dimashq significantly donated blood more frequently than those from Latakia, Homs, and Aleppo (*p* = 0.023) which is not significant. However, the reasons for blood donations did not vary between the governorates (*p* > 0.05). The higher number of blood donations every year was associated with lower DAR-5 scores (*p* = 0.020) and stress scores (*p* = 0.018).

When using chi-square with *p*-values corrected using Bonferroni, change in place of living due to war was not associated with the changes in the blood donation patterns. Although distress from war noise was significantly associated with fewer blood donations (*p* < 0.001), the increase in blood donations was associated with life being endangered by war (*p* = 0.013), which is not significant, and losing someone due to war (*p* = 0.001). Furthermore, losing someone due to war was associated with other reasons for blood donation instead of donating for obtaining a document (*p* = 0.015), while those who were distressed did not donate because of COVID-19 and it was hard to leave or reach the center more frequently (*p* = 0.002).

When using chi-square and Bonferroni corrections, fear of getting infected was associated with not donating blood, but not significantly (*p* = 0.029). Those who were worried about getting infected were less likely to donate blood to get documents when compared to others, but were significantly affected by the quarantine (*p* = 0.021). Among those who declared that monthly income adequacy cannot cover essentials, some frequently declared that they did not donate blood because of the lockdown or the center being far or hard to reach (*p* = 0.058). Smoking was significantly associated with more blood donations (*p* < 0.001), but this was not observed for shisha smoking. Donating blood or not was significantly positively correlated with age (*p* < 0.001) with a different mean age of 3 years between the two groups. Not donating blood because of the lockdown or the center being far were not associated with being endangered directly by war, losing someone close, and changing the place of living due to war (*p* > 0.05), but they were significantly associated with being distressed by war noise (*p* = 0.002).

## Discussion

Our study demonstrated that most people who did not donate blood during COVID-19 attributed this to not having a reason to do it. The study findings suggest that the main reason to donate blood in Syria, which was for obtaining a government document, was no longer necessary during COVID-19 and the lockdown. People did not have any reason to donate blood which lowered the blood product supply. The number of people who did not donate blood out of fear of COVID-19 was not statistically significant, and the number of people who declared that they only donated blood to obtain documents was 64.8%. This study also suggested that being a female, being distressed by war noise, older age, and smoking were associated with more frequent blood donations. Interestingly, losing someone due to war was associated with increased blood donation as well.

Although social distancing and lockdown were among the main reasons for the decrease in blood donations in many countries (Al Mahmasani et al., 2021), most of the participants from Syria declared that they did not donate blood simply because they did not need to and not because of COVID-19 distress or restrictions.

Surprisingly, anger and stress during the lockdown were not associated with any change in blood donations, and participants did not report their donations being affected by COVID-19. However, losing someone close or being endangered by war were associated with more blood donations. In contrast, being distressed by war noise was associated with fewer blood donations, and more were concerned about getting infected. Many of the documents in Syria, as mentioned earlier, cannot be obtained without having a blood donation certificate which we speculate was the main reason for the decrease in blood donations during the lockdown.

We hypothesize that people in Damascus and Rif-Dimashq donated blood more frequently because it is usually stricter there to donate blood to acquire required documents, whereas other governorates depend more on voluntary blood donations.

We could not find any study that directly links stress and/or anger from COVID-19 with blood donation patterns as most studies only associated COVID-19 and the lockdown without assessing the effect of the psychological aspects of COVID-19 on blood donations. Blood donation can have direct benefits on the mental health of the donors as a study found that blood donation can help manage negative emotions such as anger (Ferguson and Masser, 2018).

Globally, several measures were taken to tackle the blood shortage problem due to COVID-19 as it was proven that there was a significant reduction in blood donations compared to previous years (Al Mahmasani et al., 2021). Some medical strategies to spare blood were used to reduce the need for blood (Al Mahmasani et al., 2021). Other measures that can be taken in Syria are spreading awareness about COVID-19 and blood donation to encourage blood donation and make appointments to ensure social distancing. They were particularly important for Syrians as they rely on search engines to get information and they rarely rely on official sources (Alyousbashi and Almahayni, in press).

Donors who were exposed to a possible COVID-19 case or from a region with a high COVID-19 burden should refrain from blood donation for at least 4 weeks (Cai et al., 2020). Furthermore, pre-donation procedures should be carried out such as physical examination and temperature measuring (Cai et al., 2020).

Recruiting donors by using traditional methods and social media were the main methods used by many countries (Al Mahmasani et al., 2021). Furthermore, up-to-date and accurate data can reduce distress from COVID-19 (Luo et al., 2020). Another method used by many countries is pleading with the public to donate blood (Al Mahmasani et al., 2021), and as the people in Syria used to donate when it was necessary for them, this might be useful. Finally, effective campaigns, spreading awareness, and giving social value to blood donation were used in many countries (Al Mahmasani et al., 2021), and these should be addressed in Syria where a lack of motivation was evident. Moreover, this proves that mandatory blood donation, like many mandatory practices, fails in times of need.

## Limitations

This study was online-based and could not directly target blood donation centers that might have presented a more detailed result. It included social media users alone and excluded those who do not use it. Moreover, not having previous data from Syria is also a limiting factor. Moreover, the relatively high socioeconomic status of the participants and their relatively younger age may make it difficult to generalize the results to the entire population. Recall bias was also a factor in our study as people might not correctly recall why they donated blood a few years earlier or the number of times they donated blood. Using social media caused a bias when choosing the population as it is difficult to exactly determine the response rates. Inexperienced blood donors, in general, exhibit higher stress and anxiety before blood donations (Hoogerwerf et al., 2015). This study assessed the stress and anger and blood donation patterns over the last few weeks. Therefore, most previous biases should be minimum, particularly as people were in lockdown and had a lot of time to spend on social media in Syria.

## Conclusion

Stress and anger from COVID-19 were not associated with the reduction in blood donation practices. Although it is practical to make blood donation mandatory for obtaining documents without depending on volunteer blood donation, this is not sustainable and cannot work in crisis situations. Syria provides a unique model for studying blood donation patterns as it has suffered from 9 years of war and a 10-week full lockdown. Stress from fear of being infected was not associated with the decrease in the frequency of blood donation in a setting where people strive to get by.

## Data availability statement

The raw data supporting the conclusions of this article will be made available by the authors upon reasonable request, without undue reservation.

## Ethics statement

Ethical aspects of this research were reviewed and approved by Damascus University Deanship who represents the leading Ethical Committee at Damascus University Faculty of Medicine and that was according to the principles embodied in the Declaration of Helsinki. Informed consent was taken from participants before continuing with the survey.

## Author contributions

AK: original draft, methodology, supervising, data collection, reviewing the draft, analysis, statistics, and software. SM and AG: methodology, data collection, and reviewing the draft. OH: methodology and reviewing the draft. All authors contributed to the article and approved the submitted version.

## Footnotes

^1^Health LWwU. 5 Legit Reasons Why to Donate Blood. Available online at: https://www.unitypoint.org/livewell/article.aspx?id=d497dcef-68f3-47ac-8eef-16e5f51b9f66&5+Legit+Reasons+Why+to+Donate+Blood.
